# A Black Raspberry-Rich Diet Protects From Dextran Sulfate Sodium-Induced Intestinal Inflammation and Host Metabolic Perturbation in Association With Increased Aryl Hydrocarbon Receptor Ligands in the Gut Microbiota of Mice

**DOI:** 10.3389/fnut.2022.842298

**Published:** 2022-06-06

**Authors:** Pengcheng Tu, Liang Chi, Xiaoming Bian, Bei Gao, Hongyu Ru, Kun Lu

**Affiliations:** ^1^Department of Environmental Sciences and Engineering, University of North Carolina at Chapel Hill, Chapel Hill, NC, United States; ^2^Department of Environmental Health Sciences, University of Georgia, Athens, GA, United States

**Keywords:** black raspberry (*Rubus occidentalis*), gut microbiota, aryl hydrocarbon receptor (AHR), metabolomics, inflammation

## Abstract

Dietary modulation of the gut microbiota recently received considerable attention, and ligand activation of aryl hydrocarbon receptor (AHR) plays a pivotal role in intestinal immunity. Importantly, black raspberry (BRB, *Rubus occidentalis*) is associated with a variety of beneficial health effects. We aim to investigate effects of a BRB-rich diet on dextran sulfate sodium (DSS)-induced intestinal inflammation and to determine whether its consequent anti-inflammatory effects are relevant to modulation of the gut microbiota, especially its production of AHR ligands. A mouse model of DSS-induced intestinal inflammation was used in the present study. C57BL/6J mice were fed either AIN-76A or BRB diet. Composition and functions of the gut microbiota were assessed by 16S rRNA sequencing and comparative metagenome analysis. Metabolic profiles of host and the gut microbiome were assessed by serum and fecal metabolomic profiling and identification. BRB diet was found to ameliorate DSS-induced intestinal inflammation and host metabolic perturbation. BRB diet also protected from DSS-induced perturbation in diversity and composition in the gut microbiota. BRB diet promoted AHR ligand production by the gut microbiota, as revealed by increased levels of fecal AHR activity in addition to increased levels of two known AHR ligands, hemin and biliverdin. Accordingly, enrichment of bacterial genes and pathways responsible for production of hemin and biliverdin were found, specific gut bacteria that are highly correlated with abundances of hemin and biliverdin were also identified. BRB dietary intervention ameliorated intestinal inflammation in mice in association with promotion of AHR ligand production by the gut microbiota.

## Introduction

The gut microbiota is well-recognized for its critical functions in the immune system (1, 2), metabolic processes (3), and diverse signaling pathways (4). Mounting evidence has indicated that an imbalanced gut microbiota is highly associated with various human diseases, including inflammatory bowel disease (IBD) (5), colorectal cancer (6), obesity (7), and neurological disorders (8). Even with an increasing understanding of the association between adverse health outcomes and gut microbial patterns, the functional link between gut bacteria and the host remains elusive. Concurrently, gut microbiome-derived specialized metabolites contribute in a significant way to host physiology (3, 9, 10). For example, bacterial metabolic products that are ligands to the aryl hydrocarbon receptor (AHR) lead to effects on intestinal immune cells and mucosal barrier (11–13). Thus, production of bacterial metabolites is an important factor for health implications of gut microbial activities.

Diet emerges as an essential determinant of gut microbial structure and function (14). It is suggested that the Western diet that is rich in saturated fat and simple sugars is associated with elevated risk of metabolic diseases such as obesity, diabetes, cardiovascular diseases, and chronic inflammation (15). Alternatively, diets rich in berries, a good source of antioxidant polyphenols and soluble fiber, protect from such metabolic diseases (16). Therefore, health implications of healthy or unhealthy dietary patterns are, respectively, associated with concomitant gut microbial changes (17). In addition, dietary modulation, especially whole food-based approaches, of the gut microbiome received considerable attention due to the advantages of low toxicity profiles and high patient compliance (18). We previously characterized the gut microbiome and its metabolic profile in healthy mice with consumption of black raspberries (BRBs) which indicated its potential in functional gut microbiome modulation (19, 20). Given the perspective of whole food-based approaches coupled with health benefits of berries, there is a need to elucidate the effects of black raspberries on microbiota-associated diseases such as intestinal inflammation through the lens of gut microbiome modulation.

Intestinal inflammation is involved in development of multiple intestinal disorders including IBD. IBD, including Crohn’s disease and ulcerative colitis, is a complex inflammatory disorder of the digestive tract, which is associated with an abnormal interaction between gut bacteria and immune system (5). In particular, intestinal AHR expression is found to be significantly diminished in IBD patients (21), and increased activation of AHR is shown to suppress inflammation in mice of experimental colitis (22). AHR is a ligand-activated transcription factor that has a variety of endogenous and exogenous ligands (23, 24). Mounting evidence showed that gut microbial metabolites are an important source of endogenous AHR ligands (25). For example, tryptophan metabolites produced by gut bacteria, such as indole-3-acetate, act as AHR ligands and play a protective role in intestinal homeostasis (26, 27). Together, these observations suggested the relevance of microbiome-derived metabolites to intestinal inflammatory status by acting as AHR ligands.

Given the functional role of AHR in intestinal immunity, coupled with production of AHR ligands by gut bacteria, to modulate gut microbial production of AHR ligands is an attractive therapeutic approach to intestinal inflammation and associated diseases. Particularly, a BRB-rich diet has been consistently shown to alleviate intestinal inflammation of experimental colitis (28) and suppress colorectal cancer in mice and humans (28–30). In the present study, we used the BRB diet as an approach for gut microbiome modulation to investigate its effects on intestinal inflammation. We hypothesized that a global understanding of gut bacterial metabolites could yield insights into currently uncharacterized microbiome-derived AHR ligands that have the potential to beneficially affect host health. We first validated the ameliorating effect on intestinal inflammation and host metabolic dysfunction by the BRB diet in dextran sulfate sodium (DSS)-treated mice. We next examined diversity and composition in the gut microbial communities. Furthermore, we showed that the cecal contents of BRB-fed mice had significantly higher AHR activity in addition to enriched levels of two known AHR ligands, hemin and biliverdin. Accordingly, significantly enriched bacterial genes and pathways responsible for production of hemin and biliverdin were found in the gut microbiome of BRB-fed mice, specific gut bacteria that are highly correlated with abundances of hemin and biliverdin were also identified, suggesting the involvement of gut microbial activities in producing these AHR ligands. This study offered insights regarding microbial production of AHR ligands as an attractive therapeutic approach for intestinal inflammatory disorders *via* BRB-based dietary modulation. More importantly, additional evidence was provided on the gut microbiota-host communications through microbiome-derived metabolites especially communications through individual microbiota-derived metabolites.

## Results

### Ameliorating Effect on Dextran Sulfate Sodium-Induced Intestinal Inflammation by Black Raspberry Dietary Intervention

**FIGURE 1:**
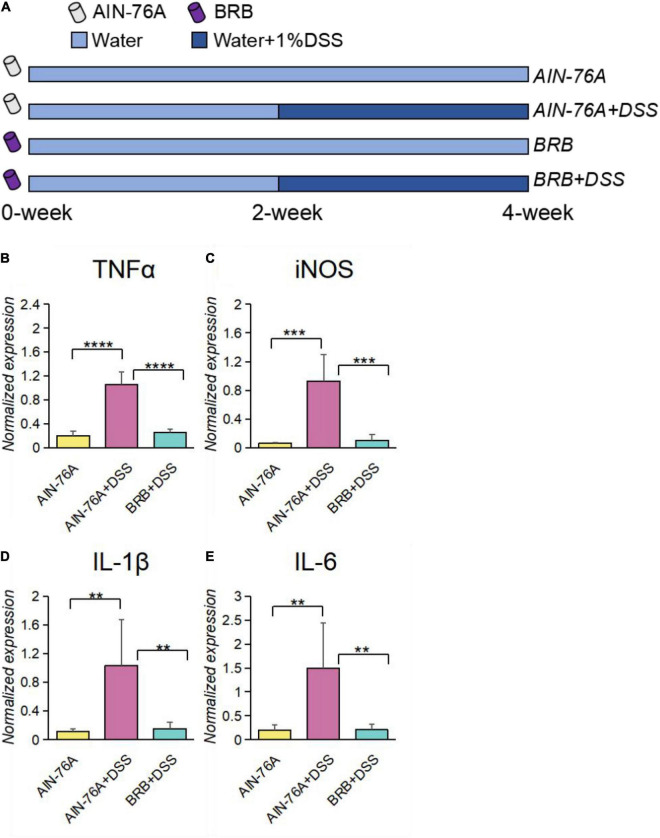
Intestinal inflammatory levels of DSS-treated mice (AIN-76 + DSS) were significantly higher than that of controls (AIN-76A), which was alleviated in mice with BRB dietary intervention (BRB + DSS). **(A)** Experimental design; 40 mice were randomly assigned into 4 groups: AIN-76A, AIN-76A + DSS, BRB, BRB + DSS; mice from each group were fed the according diet, and 1% DSS was added in their drinking water for 2 weeks of mice with DSS treatment. Gene expression of inflammatory markers, including TNFα **(B)**, iNOS **(C)**, IL-1β **(D)**, and IL-6 **(E)** were significantly increased by DSS treatment, and significantly inhibited by BRB dietary intervention. (AIN-76A, *n* = 5; AIN-76A + DSS, *n* = 4; BRB + DSS, *n* = 5; 5 mice were randomly selected per group, and for AIN-76A + DSS group, one mouse was excluded for poor quality of the extracted RNA; data were expressed as mean with SEM, one-way ANOVA followed by Tukey’s test; **p < 0.01 ***p < 0.001 ****p < 0.0001).

To determine the effects of BRB dietary intervention on intestinal inflammation, mice were given either AIN-76A diet (Control diet) or BRB diet (Control diet with 10% freeze-dried BRB powder; details of diet preparation and characterization are described in section “Materials and Methods”) in addition to plain water for 2 weeks, and 1% DSS was then added to the drinking water of DSS-treated groups for another 2 weeks to induce intestinal inflammation as described in Figure 1A. As shown in Figures 1B–E, gene expression of pro-inflammatory molecules was significantly increased by DSS treatment, and which was significantly suppressed by BRB diet. Specifically, DSS treatment significantly induced TNFα and iNOS expression in the colon tissue of mice, and such pro-inflammatory effects were significantly suppressed if concurrently given BRB diet (Figures 1B,C); we also observed similar trends in the expression of IL-6 and IL-1β (Figures 1D,E). The suppressed expression of pro-inflammatory molecules indicated the ameliorating effects on intestinal inflammation by BRB dietary intervention, which is consistent with the results of previous studies (28).

### Protection by Black Raspberry Dietary Intervention From Host Metabolic Perturbation

**FIGURE 2:**
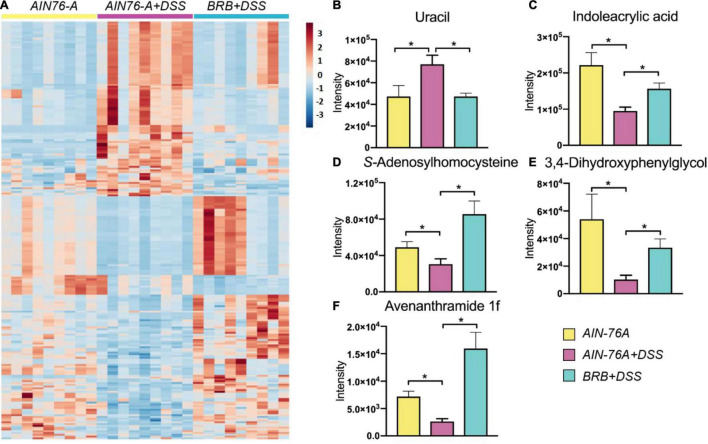
Metabolic profiling of mouse plasma indicated that DSS treatment-induced host metabolic perturbation, at least in part, suppressed by BRB dietary intervention. **(A)** Heat map shows a clear restoration pattern of metabolic features by BRB dietary intervention. Plasma metabolites, including uracil **(B)**, indoleacrylic acid **(C)**, S-Adenosylhomocysteine **(D)**, 3,4-Dihydroxyphenylglycol **(E)**, and avenanthramide 1f **(F)**, are significantly changed by DSS treatment, and significantly suppressed by BRB dietary intervention. (*n* = 9; data were expressed as mean with SEM, Welch’s two sample *t*-test was conducted AIN-76A vs. DSS, DSS vs. DSS + BRB, respectively; *p < 0.05).

We examined host plasma metabolome profiles to further investigate host response using an untargeted metabolomics approach. Principal component analysis (PCA) showed that the metabolome profiles of *AIN76-A diet*, *AIN76-A diet* + *DSS* and *BRB diet* + *DSS* groups (Supplementary Figure 1A). Moreover, 788 significantly altered features were discovered between *AIN-76A diet* and *AIN-76A diet* + *DSS* groups, and the comparison between *AIN-76A diet* + *DSS* and *BRB* + *DSS* groups revealed 846 significantly altered features, which shared 234 features with the former 788 features (Supplementary Figure 1B). Hierarchical clustering heat map constructed using the intensities of these 234 shared features showed consistent patterns within individual groups (Figure 2A). Metabolic perturbations induced by DSS treatment were largely suppressed by BRB dietary intervention, supporting the ameliorating effects of BRB on host inflammatory status. We next conducted identification of those 234 shared features. A number of metabolites (Supplementary Table 1) were identified based on accurate mass, MS/MS spectra and database matching. Figures 2B–F lists a few representative metabolites that were significantly perturbed by DSS, and the perturbation was then significantly suppressed by BRB dietary intervention. Several bacteria-derived metabolites associated with inflammation were significantly changed by DSS treatment but suppressed by BRB diet, for instance, uracil (Figure 2B) and indoleacrylic acid (Figure 2C). Previous studies showed that bacterial production of uracil favors intestinal inflammation (31), and indoleacrylic acid produced by the gut bacteria is protective against intestinal inflammation (32), which is consistent with their changes in the present study. Together the data indicated that the host metabolic perturbation induced by DSS treatment were partially suppressed by BRB dietary intervention, confirming the protective role of BRB diet in DSS-induced intestinal inflammation.

### Perturbation and Restoration of Diversity and Composition in Gut Microbial Communities

**FIGURE 3:**
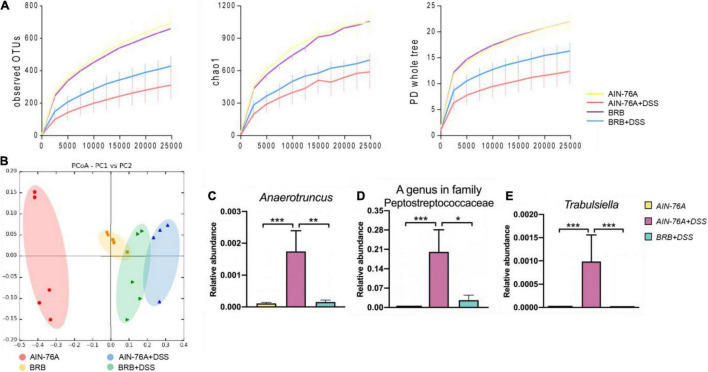
Dextran sulfate sodium (DSS)-induced perturbation in gut microbial communities were partly rescued by BRB dietary intervention. **(A)** Alpha rarefactions using PD whole tree, observed OTUs, and chao1 metrics, the *x*-axis is sequencing depth; compared to control subjects, DSS induced significant reduction in alpha diversity, which was partly restored by BRB dietary intervention. **(B)** Principle coordinate analysis presents comparison of the mouse gut microbiota of different groups (AIN-76A, BRB, AIN-76A + DSS, BRB + DSS). Relative abundances of inflammation-associated gut bacterial genera including *Anaerotruncus* **(C)**, A genus in family *Peptostreptococcaceae* **(D)**, and *Trabulsiella* **(E)**. (AIN-76A, *n* = 10; AIN-76A + DSS, *n* = 9; BRB + DSS, *n* = 10; *p < 0.05), **p < 0.01, ***p < 0.001.

We next analyzed 16S rRNA sequencing data to investigate alterations in diversity and composition of the mouse gut microbiota induced by DSS treatment with or without BRB dietary intervention. Alpha diversities were measured and compared in the overall microbial community using observed OTUs, chao1, and PD whole tree metrics as shown in Figure 3A. Generally, DSS treatment reduced alpha diversities regardless of diet types, however, the reduction was attenuated in mice with BRB diet compared to that with control diet. Moreover, principal coordinate analysis (PCoA) shows that *AIN76-A diet* + *DSS* group was well separated from *AIN76-A diet* group; *AIN76-A diet* + *DSS* and *BRB diet* + *DSS* groups were also different (Figure 3B). Diversity analysis suggested that, with BRB dietary intervention, reduction in gut microbial species richness was partially restored with perturbation to a relatively smaller extent. In addition, DSS treatment substantially changed the gut microbial composition with a number of significantly altered gut bacteria. Figures 3C–E and Supplementary Figure 2 showed bacterial genera that were significantly altered by DSS treatment, and the alteration was significantly suppressed by BRB dietary intervention. Particularly, increased levels of *Anaerotruncus*, *Trabulsiella*, and *Peptostreptococcaceae* were previously found in fecal samples of patients with colorectal cancer and Crohn’s disease (33, 34), which is consistent with their changes in the present study. Taken together, the data suggested that DSS-induced perturbation in gut microbial diversity and composition was at least partly suppressed by BRB dietary intervention.

### Gut Microbial Metabolome Featuring Abundant Aryl Hydrocarbon Receptor Ligands in Mice With Black Raspberry Diet Treatment

**FIGURE 4:**
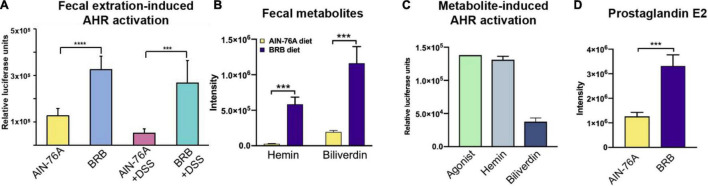
Mice with BRB dietary intervention had increased levels of intestinal AHR activity resulting from higher levels of AHR ligands. **(A)** Levels of cecal AHR activity of different groups (AIN-76A, BRB, AIN-76A + DSS, BRB + DSS; *n* = 7; one-way ANOVA followed by Tukey’s test; ****p < 0.0001; ***p < 0.001). **(B,C)** The levels of AHR agonists, hemin and biliverdin, were significantly higher in the gut microbiome of mice on BRB diet compared to that on control diet (*n* = 10; Welch’s two sample *t*-test; ***p < 0.001). **(D)** PGE2, product of AHR-activated anti-inflammatory pathway, significantly increased in mice on BRB diet compared to controls. (*n* = 10; Welch’s two sample *t*-test.

Accumulating evidence suggested the link between AHR activity and intestinal inflammation (21, 22, 35). To investigate the mechanism underlying the ameliorating effect of BRB dietary intervention, we examined the AHR activity in the gut microbiome contents of mice on different diets. Figure 4A showed the AHR activation of cecal contents by AHR reporter assay. The levels of AHR activation of *AIN-76A* + *DSS* group were significantly lower than that of *AIN-76A* group. In contrast, mice on BRB diet had significantly increased levels of AHR activation compared to mice on control diet regardless of DSS treatment (*AIN-76A* vs. *BRB* + *DSS*; *AIN-76A* + *DSS* vs. *BRB* + *DSS*), suggesting that BRB diet leads to increased levels of AHR ligands in cecal contents. We next identified specific metabolites that are AHR ligands, we conducted untargeted metabolomics on fecal contents to identify the differential metabolites of the gut microbiome contents between mice on BRB diet or control diet. As shown in Figure 4B, the relative abundances of hemin and biliverdin, that are previously reported AHR ligands, were found to be significantly higher in mice fed BRB diet compared to mice fed control diet (24). Specifically, hemin was increased by 20-fold and biliverdin was increased by sixfold. Meanwhile, we verified the AHR agonist activities of these two metabolites using TCDD as positive control (Figure 4C). In addition, it is previously reported that AHR activation ameliorated DSS-induced colitis through prostaglandin E2 (PGE2) production in the colon (36). Accordingly, we observed increased levels of PGE2 in fecal samples with higher AHR activity (Figure 4D), further supporting the role of AHR activity in ameliorating the inflammation. Taken together, these data indicated that BRB diet-modulated gut microbial metabolome had higher AHR-activating capability as well as increased levels of AHR ligands including hemin and biliverdin, which probably accounted for the ameliorating effects on intestinal inflammation.

### Enrichment of Hemin and Biliverdin Probably Originated From Gut Bacterial Metabolic Activities

**FIGURE 5:**
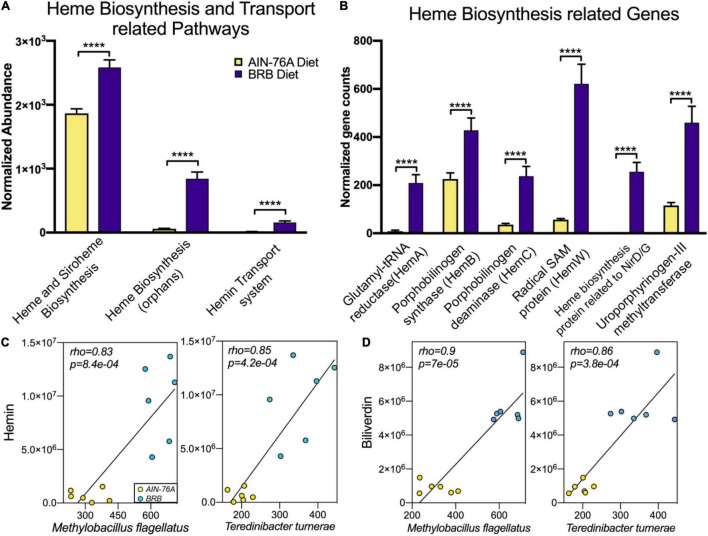
BRB-modulated mouse gut microbiome had enriched bacterial pathways **(A)** and genes **(B)** involved in production of heme-related metabolites, which probably contributed to intestinal levels of hemin and biliverdin (*n* = 6; DESeq2 FDR adjusted *p*-value). **(C,D)** Scatter plots illustrating strong statistical association between relative abundances of gut bacterial species and mass spectrum intensities of hemin **(C)** and biliverdin **(D)**, two bacterial species *Methylobacillus flagellates* and *Teredinibacter turnerae* are significantly correlated with both hemin and biliverdin (*n* = 6; rho > 0.8; *p* < 0.001), ****p < 0.0001.

To determine whether the gut microbiota was a possible source for hemin and biliverdin, we compared microbial metagenome from mice on control or BRB diets. We identified a variety of bacterial pathways and genes that are responsible for heme biosynthesis and transportation, which were significantly more abundant in BRB diet-modulated gut microbiome (Figures 5A,B). To further explore the relationship between specific gut bacteria and intestinal AHR ligands, we conducted functional correlation between the gut microbial species and metabolites. Strong correlations were identified between relative abundances of gut bacterial species and intensities of hemin and biliverdin (rho > 0.8; *p* < 0.001). Specifically, bacterial species *Methylobacillus flagellates*, *Teredinibacter turnerae*, *Cyanothece* sp. *PCC 7424*, and *Aromatoleum aromaticum* are found to be highly correlated with both hemin and biliverdin (Figures 5C,D and Supplementary Figure 3). In addition, *Methylobacillus flagellates* and *Teredinibacter turnerae* were previously reported to possess the sets of genes that are involved in heme synthesis and transportation (37, 38), further supporting the involvement of bacterial metabolic activities in hemin and biliverdin. These data provided evidence on the possibility that increased levels of intestinal AHR ligands could be derived from the gut microbiota.

## Discussion

**FIGURE 6:**
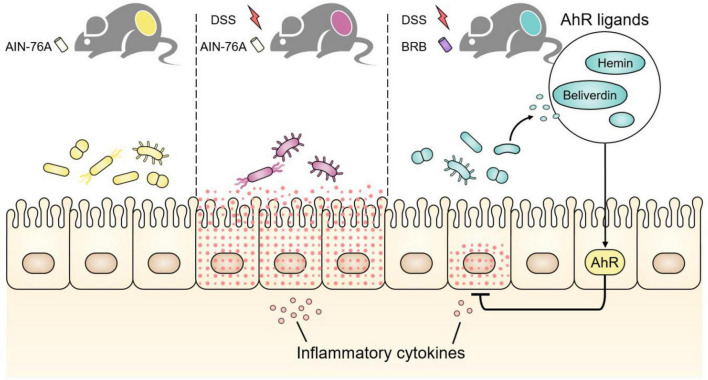
Dextran sulfate sodium (DSS) treatment caused gut microbial disruption in addition to severe intestinal inflammation, which was ameliorated if mice were concurrently fed BRB diet. BRB dietary intervention increased intestinal AHR activity by modulating the gut microbiota and promoting production of microbiome-derived AHR ligands, for instance, hemin and biliverdin. Intestinal AHR activation contributed to alleviation of inflammatory response and restoration of host metabolic perturbation *via* diverse mechanisms including production of PGE2.

We used a BRB-rich diet to study the involvement of the gut microbiota and its modulation in intestinal inflammation. The data clearly showed that BRB dietary intervention reduced DSS-induced inflammation in mouse colon and increased intestinal levels of AHR activity. More importantly, the elevated AHR activity probably originated from BRB-modulated production of AHR ligands by gut bacteria. Figure 6 shows the proposed mechanism underlying the ameliorating effects on intestinal inflammation. Compared to mice on control diet, mice on BRB diet suffered less severe intestinal inflammation from DSS treatment. Furthermore, DSS-induced perturbation in the gut microbiota was partially suppressed. Metabolic activities of gut bacteria were modulated by BRB consumption, which leads to increased intestinal levels of AHR ligands hence enhanced intestinal AHR activation, contributing to amelioration of inflammation and restoration of host metabolic profiles *via* diverse mechanisms including production of PGE2. These findings may offer novel insights regarding modulation of the gut microbiota and its metabolites as a new mechanism of beneficial health effects from BRB consumption. More importantly, dietary effects on gut bacterial production of AHR ligands provided additional evidence on the intertwined relationship among diets, the gut microbiome and host health.

Accumulating evidence suggested the association between IBD and the gut microbiota although other factors also play important roles such as genetic and environmental elements (39, 40). For instance, reduction in alpha diversities of the gut microbiota is highly associated with IBD development (41). Furthermore, it is well demonstrated that AHR plays a protective role in IBD *via* modulation of intestinal immune response and barrier integrity (22, 42). AHR can be activated by a range of ligands originating from dietary components or gut microbial metabolic activities (25). Microbiome-derived metabolites are an important source of intestinal AHR ligands (25). It is suggested that, perturbation in the gut microbiome associated with intestinal inflammation reduces production of microbiome-derived AHR ligands, leading to decreased AHR ligand availability in the intestine, hence decreased intestinal AHR activity. Perturbed intestinal AHR activity contributes to alterations in intestinal immune response and barrier functions that further amplify the gut microbiome perturbation in a vicious cycle, favoring chronic intestinal inflammation and consequently leading to IBD (43). Thus, restoration of the perturbed gut microbiome especially its production of AHR ligands will probably break the vicious cycle and ameliorate the intestinal inflammation. In the present study, we assessed the severity of intestinal inflammation and AHR activity in gut microbiome contents (Figures 1, 4); and we found that the inflammation was ameliorated in association with promotion of AHR ligand production by the gut microbiota (Figure 4), suggesting the protective role of AHR activation against intestinal inflammation. More importantly, enriched abundances of bacterial pathways and genes related to AHR ligand production in addition to strong correlations between bacterial species and AHR ligands suggested the involvement of the gut microbiota. Here, we have identified one potential contributor, namely, microbiome-derived AHR ligands to intestinal inflammatory status.

Aryl hydrocarbon receptor is a member of the basic helix–loop–helix–(bHLH) superfamily of transcription factors. A myriad of genes are regulated by AHR, including those encoding xenobiotic metabolizing enzymes, such as Cytochrome P450 1A1 (*Cyp1a1*) (44). Besides its toxicological involvement, AHR activation upon binding to a wide array of endogenous and exogenous ligands, leads to numerous key host physiological functions in intestinal barrier function and intestinal immune cells (43). AHR is expressed by intestinal epithelial cells (IEC) in the intestine. IECs regulate AhR ligand availability to intestinal immune cells, and their AHR signaling is pivotal in the regulation of mucosal intestinal immune responses (43). IL-22 production is mediated by AHR, which is involved in mucosal wound-healing and production of antimicrobial peptides (AMPs) (21). In addition, AHR activation is also involved in intestinal expression of IL-10 and Reg3g, which are essential for intestinal homeostasis (43). The gut microbiota communicates with the host through production of functional metabolites (9). Cellular functions and host physiology can be directly altered by bacterial metabolic products. For example, many bacterial metabolites act as signaling molecules (4). Short-chain fatty acids (SCFAs) produced by gut bacteria regulate intestinal immune functions through binding to the G-protein-coupled receptors (GPCRs) (45). Likewise, tryptophan metabolites produced by gut bacteria can bind to AHR affecting gut immune responses (13). Previous studies on microbiome-derived AHR ligands mostly focused on tryptophan derivatives (12, 25). However, bacteria are known to possess the sets of genes and functional pathways in production of heme-related molecules (46–49), which are also recognized as AHR ligands (23, 24). The present study found significantly increased levels of hemin and biliverdin in gut microbiome contents in concert with enriched bacterial pathways and genes involved in their biosynthesis (Figures 4, 5), adding more members to the reservoir of microbiome-derived AHR ligands.

Intestinal inflammation is associated with host metabolic perturbation (50), which is partially attributed to differential gut microbial activities (51). A relevant animal model may offer mechanistic insights and explore biomarkers regarding intestinal inflammation and the gut microbiota. We observed significantly perturbed metabolite fingerprints in plasma of mice with intestinal inflammation, and the perturbation was largely suppressed if mice were concurrently given BRB diet. It is of necessity to point out that indeed there are some features in the heat map that are not fully consistent with the general pattern of restoration. Specifically, some of the restored metabolites are microbiome-derived and inflammation-relevant, which makes them great biomarker candidates of gut microbiota-related inflammation. For example, uracil levels in plasma were significantly increased by DSS treatment and the elevation was largely prevented by BRB dietary intervention (Figure 2B). Uracil may serve as a specific indicator of bacteria-related intestinal inflammation because bacterial production of uracil activates intestinal innate immune cells and leads to inflammatory response (31). Likewise, DSS reduced levels of plasma indoleacrylic acid, which was suppressed if mice were fed BRB diet (Figure 2C). Indoleacrylic acid can be produced by gut bacteria and is protective against intestinal inflammation (32). Moreover, we also discovered that S-Adenosylhomocysteine (SAH) decreased in mice of DSS treatment (Figure 2D), which is in accordance with the previous report (51). There is growing appreciation of the possible association between mucosal DNA methylation and colitis in humans (52, 53). Decreased levels of SAH may indicate interrupted methylation because SAH is generated when S-Adenosylmethionine (SAM), the methyl donor, loses the methyl group. In addition, naturally occurring phenolic compounds such as 3,4-dihydroxyphenylglycol (Figure 2E) and avenanthramides (Figure 2F) with demonstrated anti-inflammatory effects were observed to be upregulated in mice on BRB diet, indicating a protective role of BRB possibly resulting from its natural components (54, 55).

Ligand activation of AHR is an inviting therapeutic approach for intestinal inflammation; and dietary modulation of the gut microbiota received increasing attention (22, 56). Several recent studies have focused on the provision of AHR ligands from dietary components (42, 57), here we demonstrated that BRB-modulated gut microbiome contents had elevated AHR activity (Figure 4). While our data emphasize the gut microbiota could be a major contributor to intestinal AHR activity, it remains to be determined whether components in BRB are direct AHR ligands or precursors. Moreover, it is very likely that bacterial metabolites besides hemin and biliverdin are AHR ligands. Future studies on the search and identification of microbiome-derived AHR ligands and their potential in treating intestinal inflammation are warranted. Although whole food-based approaches to modulate the gut microbiome has many advantages, the complexity of functional components in BRB hinders characterization of actual effective components. Therefore, to modulate the gut microbiome using specific compounds in BRB to reproduce similar effects is also warranted.

Several limitations are associated with this study. The major goal of the present study was to better understand the role that the gut microbiome plays in the anti-inflammatory effects of BRB. Thus, a relatively low concentration of DSS administration was used to induce low-grade, reversible intestinal inflammation instead of a colitis model. In fact, we did not observe any significant colitis by H and E staining in mice given DSS, probably due to the low dose of DSS. Therefore, our data may only be interpreted in context of intestinal inflammation but not colitis. For analysis of gut microbial composition, a relatively small sample size was included in the present study, a larger sample size would definitely help to delineate the microbial community more accurately. In addition, we only conducted the comparison between AIN-76A and BRB groups in investigation of the involvement of the gut microbiome in intestinal AHR activity, with the major goal of demonstrating that altered microbial metabolites such as AHR ligands are associated with gut microbiome modulated by BRB diet. Also, mounting evidence has shown sex differences regarding activities and functions of the gut microbiome responding to external factors (58–61) as well as host health conditions (62). A single sex of mice was used for the present study, therefore, sex-specific influences regarding changes in the gut microbiome responding to the BRB diet and DSS treatment awaits future studies.

Intestinal inflammation most likely involves not just one mechanism but rather a complex interplay of genetic, environmental and microbial factors. Our study provided evidence on the role of microbiome-derived AHR ligands. Improved intestinal inflammatory status, host metabolic profiles along with restored gut microbiota indicated protective effects of BRB dietary intervention. Importantly, these effects are relevant to increased microbial production of AHR ligands. In addition to offering a potential mechanism of the anti-inflammatory effects from BRB consumption, this study indicated the gut microbiota as a source of intestinal AHR ligands, which provided new thoughts on the development of therapeutic interventions for intestinal inflammatory disorders.

## Materials and Methods

### Study Approval

The study protocol (NO: A2013 06-033-Y3-A3) was approved by the University of Georgia Institutional Animal Care and Use Committee. All methods were performed in accordance with the relevant guidelines and regulations. All efforts were made to minimize animal suffering.

### Diet Preparation

Custom purified American Institute of Nutrition (AIN)-76A animal diet (Dyets, Inc., Bethlehem, PA, United States) was used as the control diet. BRB diet used for dietary intervention was prepared as described in Oghumu, et al. (63). Briefly, whole ripe BRB (*Rubus occidentalis*) of the Jewel variety were harvested from a single farm in Southern Ohio, and then were freeze-dried and ground into powder. BRB powder was stored at −20°C until being incorporated into AIN-76A animal diet pellets by 10% w/w concentration at the expense of cornstarch. The diets were stored at 4°C until being fed to mice. Mice of control diet groups were fed AIN-76A diet, mice of dietary intervention groups were fed BRB diet. Composition of control and BRB diets can be found in the previous report (64). Its preparation was standardized to ensure consistency and reproducible results. The BRB diet used in the present study was previously used in a large number of studies for chemopreventive effects as well as microbiome modulation (19, 20, 65–69). Further details regarding the BRB diet were previously discussed and reviewed (70).

### Mice

Specific-pathogen-free C57BL/6 mice (Male; 8 weeks of age; Jackson Laboratories, Bar Harbor, ME, United States) were housed in the animal facility of University of Georgia. Plain water and standard pelleted rodent diet *ad libitum* were provided for 1 week for their acclimation. The environmental conditions were maintained as 22°C temperature, 40–70% humidity, and a 12:12 h light:dark cycle. After 1 week of acclimation, the mice were then randomly assigned to 4 groups (Figure 1A; AIN-76A, AIN-76A + DSS, BRB, BRB + DSS; *n* = 10 per group). Their food was provided with AIN-76A or BRB diets accordingly. After 2 weeks of special diets, 1% DSS in drinking water was administered to mice of DSS treatment groups for another 2 weeks. A lower concentration (1%) of DSS administration was used compared to the concentration used in the colitis model (3%) to induce relatively low-grade, reversible intestinal inflammation. Regular monitoring for health conditions was done twice a week. Fecal samples were collected individually before sacrifice; Animals were fasted overnight before sacrifice. Plasma, cecal contents, and colon tissues were collected during necropsy and colon tissues were treated with RNAlater (Thermo Fisher Scientific). All samples were kept at −80°C until further analysis. The mice were treated humanely and with regard for alleviation of suffering.

### Quantitative RT-PCR

Colon RNA was extracted using RNeasy Mini kit (Qiagen, Valencia, CA, United States) according to manufacturer’s instructions. Then RNA was processed with DNA-free™ DNA Removal Kit (Thermo Fisher Scientific) to remove DNA contamination. RNA quality and concentration were determined using an Agilent TapeStation (Agilent Technologies). Reverse transcription was performed with iScript™ Reverse Transcription Supermix (Bio-Rad Laboratories, CA, United States) according to manufacturer’s instructions. qPCR was performed with the SsoAdvanced™ Universal SYBR^®^ Green Supermix (Bio-Rad) and primers listed in Supplementary Table 2. The reactions were run on a Bio-Rad CFX96 Touch™ Real-Time PCR Detection System using the protocol as previously described in Bian et al. (71) (Bian, Tu et al. 2017). Results were analyzed by the ΔΔCt method of CFX manager software (Bio-Rad) using *Gapdh* as the internal control.

### 16S rRNA Gene Sequencing and Analysis

16S rRNA gene sequencing was performed as previously described in Chi et al. (72). Briefly, microbial DNA was extracted from mouse fecal pellets using PowerSoil DNA isolation kit according to manufacturer’s instructions. Then the DNA was amplified using 515 (5′-GTGCCAGCMGCCGCGGTAA) and 806 (5′-GGACTACHVGGGTWTCTAAT) primers targeting the V4 regions of 16S rRNA gene in bacteria (73). Individual samples were normalized, barcoded and finally pooled for the construction of the sequencing library, then sequenced using the Illumina MiSeq (500 cycles v2 kit) in the Georgia Genomics Facility of University of Georgia. Paired reads were assembled using Geneious 8.15 (Biomatters, Auckland, New Zealand). Operational taxonomic unit (OTU) picking and diversity analysis were conducted using Quantitative Insights into Microbial Ecology (QIIME, version 1.9.1).

### Reporter Assay for Aryl Hydrocarbon Receptor Activation

Aryl hydrocarbon receptor activation was measured using a commercially available Reporter Assay System (INDIGO Biosciences, Inc., State College, PA, United States). Mouse cecal samples were suspended in PBS (100 mg/mL), centrifuged at 5,000 rpm for 15 min at 4°C, and then filtered with 0.2 mm filters (VWR, Fontenay-sous-Bois, France) as described in the previously study (13). Cecal extraction was diluted (1:10) with Compound Screening Medium (CSM) supplied in the reporter assay kit. Hemin and biliverdin hydrochloride (Sigma-Aldrich) were dissolved in DMSO and diluted with CSM to a concentration of 50 mM. Potent AHR agonist 2,3,7,8-tetrachlorodibenzop-dioxin (TCDD) was used as the positive control.

### Metagenomics Sequencing

Shotgun metagenomic sequencing was performed as previously described in Chi et al. (72). Briefly, fecal DNA (10 ng/μL) was fragmented using the Bioruptor UCD-300 sonication device. The Kapa Hyper Prep Kit was applied to construct the sequencing library according to manufacturer’s instructions. The quantification of DNA was performed using the Qubit 2.0 Fluorometer. The sequencing was performed using the Illumina NextSeq High Output Flow Cell (300 Cycles; PE150) in the Georgia Genomics Facility of University of Georgia. The MG-RAST metagenomics analysis sever (version 4.0.3)^1^ was applied for automatic functional analysis of metagenomes using the Subsystems database (74).

### Metabolomics Profiling

For fecal samples, 20 mg feces and 50 mg glass beads (Sigma-Aldrich, MO, United States) were added to 400 μL cooled methanol solution (methanol: water 1:1), followed by homogenizing using a TissueLyser (Qiagen) for 15 min at 50 Hz. The supernatant was collected after centrifuging for 10 min at 1,2000 rpm, dried up in a speed vacuum (Thermo), and then resuspended for injection. For plasma samples, 80 μL cooled methanol was added to 20 μL plasma. After incubation for 30 min at −20°C, the samples were centrifuged for 10 min at 12,000 rpm. The supernatant was collected after centrifuging for 10 min at 1,2000 rpm, dried up in a speed vacuum, and then resuspended.

Liquid Chromatograph-Mass Spectrometer (LC-MS) analysis was performed on a quadrupole-time-of-flight (Q-TOF) 6550 mass spectrometer (Agilent Technologies, Santa Clara, CA, United States) with an electrospray ionization source. The mass spectrometer was interfaced with an Agilent 1290 Infinity II UPLC system. Metabolites were analyzed in the positive mode over a m/z range of 50–1000 with a C18 T3 reverse-phased column (Waters Corporation, Milford, MA, United States). The XCMS Online sever (version 3.5.1)^2^ was applied for peak picking, alignment, integration, and extraction of the peak intensities. Relative abundances of metabolites were indicated by peak intensities. A two-tailed Welch’s *t*-test was used for the assessment of differentiated metabolite features. MS/MS data were generated on the Q-TOF for the identification of differentiated metabolites. The softwares of MS-DIAL (version 2.90) (75) and MS-FINDER (version 2.40) (76) were used for the identification of metabolites based on the MS/MS spectrum.

### Statistical Analysis of Data

Unless otherwise indicated, all results are expressed as mean values with standard deviation (^****^p < 0.0001; ^***^p < 0.001; ^**^p < 0.01; *p < 0.05; N.S. *p* > 0.05). Statistical differences in gene expression of inflammatory markers and AHR activation were calculated by one-way ANOVA followed by Tukey’s test. Differences in gut bacterial abundances were assessed by a non-parametric test *via* Metastats (77). Two-tailed Welch’s *t*-test was used to analyze metabolites that differed in abundance between groups corrected for the FDR. The metagenomics sequence count data for functional analysis were processed using DESeq2 (78) for statistics analysis adjusted for multiple testing of FDR. Also, alpha rarefaction and PCoA were used to assess diversities in the gut microbial communities. PCA and hierarchical clustering algorithm were used to visualize the comparison of metabolite profiles. The correlation matrix between gut bacterial species and metabolites was generated using Pearson’s correlation coefficient.

## Data Availability Statement

The datasets presented in this study can be found in online repositories. The names of the repository/repositories and accession number(s) can be found in the article/Supplementary Material.

## Ethics Statement

The animal study was reviewed and approved by the University of Georgia Institutional Animal Care and Use Committee.

## Author Contributions

KL and HR conceived the project. PT, XB, BG, and LC conducted the experiments on animals and other lab work. PT and LC conducted the bioinformatics analysis. PT, KL, and HR wrote the manuscript. All authors read and approved the final manuscript.

## Conflict of Interest

The authors declare that the research was conducted in the absence of any commercial or financial relationships that could be construed as a potential conflict of interest.

## Publisher’s Note

All claims expressed in this article are solely those of the authors and do not necessarily represent those of their affiliated organizations, or those of the publisher, the editors and the reviewers. Any product that may be evaluated in this article, or claim that may be made by its manufacturer, is not guaranteed or endorsed by the publisher.

## Footnotes

1
http://metagenomics.anl.gov

2
https://xcmsonline.scripps.edu
