# Supplementary material for: A Black Raspberry-Rich Diet Protects From Dextran Sulfate Sodium-Induced Intestinal Inflammation and Host Metabolic Perturbation in Association With Increased Aryl Hydrocarbon Receptor Ligands in the Gut Microbiota of Mice
Source: Front Nutr. 2022 Jun 6;9:842298. doi: 10.3389/fnut.2022.842298 (PMC9208328; doi:10.3389/fnut.2022.842298)
Supplement: Supplementary file 1 [file Table_1.DOCX]

***Supplementary Material***

**A black raspberry-rich diet protects from dextran sulfate sodium-induced intestinal inflammation and host metabolic dysbiosis in association with increased aryl hydrocarbon receptor ligands in the gut microbiota of mice**

Pengcheng Tu^1†^, Liang Chi^1†^, Xiaoming Bian^2^, Bei Gao^2^, Hongyu Ru^1^, and Kun Lu^1*^

1. Department of Environmental Sciences and Engineering,

University of North Carolina at Chapel Hill, Chapel Hill, NC, 27599

2. Department of Environmental Health Sciences,

University of Georgia, Athens, GA, 30602

^†^ Contributed Equally

^*^ Corresponding Author

Kun Lu, PhD

Department of Environmental Sciences and Engineering

University of North Carolina at Chapel Hill, Chapel Hill, NC, 27599

Tel: 919-966-7337

Email: [kunlu@unc.edu](mailto:kunlu@unc.edu)


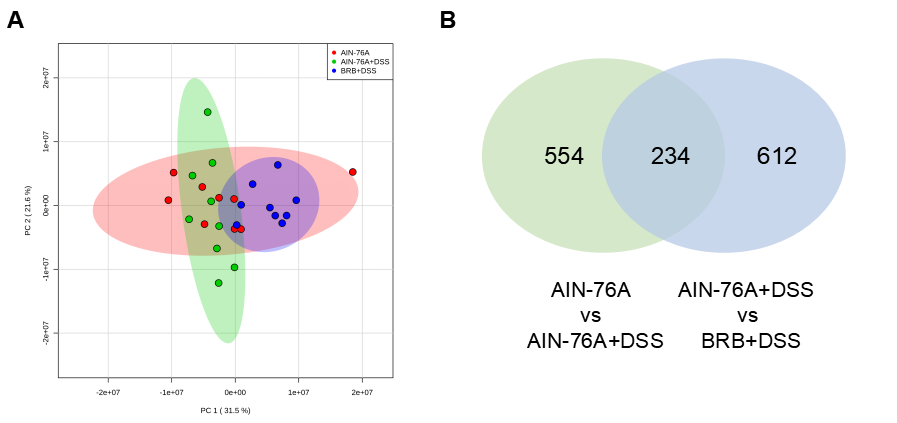


**Fig. S1.** **A** The visualization of metabolic profiles in plasma of mice from different groups by PCA; DSS-induced differences in mouse metabolic profiles disappeared if the mice were concurrently fed BRB diet instead of control diet. **B** Venn diagram of the comparisons of differentiated metabolites between groups. (n=9)

**Table S1.** List of serum metabolites that are significantly perturbed by DSS treatment and significantly restored by BRBs (p<0.05).

| Metabolite | m/z | RT(min) | Precursor type | AIN-76A+DSS/AIN-76A^*^ | BRB+DSS/AIN-76A+DSS^*^ |
| --- | --- | --- | --- | --- | --- |
| (S)-3-Hydroxyisobutyric acid | 127.0363 | 2.3 | [M+Na]+ | 1.6 | 0.6 |
| 2-Aminoheptanoate | 146.1173 | 4.8 | [M+H]+ | 18.1 | 0.2 |
| 2-Oxoarginine | 174.0878 | 1.4 | [M+H]+ | 0.6 | 2.1 |
| 3,4-Dihydroxyphenylglycol | 171.0621 | 2.9 | [M+H]+ | 0.2 | 3.3 |
| 3-Hydroxyanthranilic acid | 154.0473 | 3.0 | [M+H]+ | 2.7 | 0.3 |
| 9-Decenoylcarnitine | 314.2323 | 8.4 | [M+H]+ | 1.2 | 0.6 |
| Avenanthramide 1f | 314.1091 | 1.6 | [M+H]+ | 0.4 | 6.0 |
| Casuarine 6-alpha-D-glucoside | 368.1530 | 5.0 | [M+H]+ | 0.5 | 1.8 |
| Cyclohexylamine | 100.1113 | 4.0 | [M+H]+ | 5.9 | 0.2 |
| Furanone A | 85.0289 | 1.5 | [M+H]+ | 1.6 | 0.6 |
| Indoleacrylic acid | 188.0741 | 5.0 | [M+H]+ | 0.4 | 1.6 |
| N-(1-Deoxy-1-fructosyl)leucine | 294.1537 | 3.9 | [M+H]+ | 0.6 | 2.8 |
| N-(1-Deoxy-1-fructosyl)methionine | 312.1104 | 1.7 | [M+H]+ | 0.4 | 5.3 |
| N-(1-Deoxy-1-fructosyl)phenylalanine | 328.1383 | 4.5 | [M+H]+ | 0.7 | 2.7 |
| N-(1-Deoxy-1-fructosyl)tyrosine | 344.1333 | 2.2 | [M+H]+ | 0.7 | 2.7 |
| N-(1-Deoxy-1-fructosyl)valine | 280.1369 | 1.6 | [M+H]+ | 0.6 | 2.9 |
| Nigellic acid | 281.1396 | 1.6 | [M+H]+ | 0.5 | 2.8 |
| Proline betaine | 144.1020 | 1.2 | [M+H]+ | 0.5 | 1.9 |
| S-Adenosylhomocysteine | 385.1360 | 1.7 | [M+H]+ | 0.6 | 2.8 |
| Serotinose | 313.1136 | 1.6 | [M+H]+ | 0.4 | 5.6 |
| Succinyladenosine | 384.1147 | 4.7 | [M+H]+ | 0.6 | 1.9 |
| Tocopheronic acid | 295.1565 | 3.1 | [M+H]+ | 0.6 | 2.6 |
| Uracil | 113.0310 | 2.0 | [M+H]+ | 1.6 | 0.6 |
| z-Clausenamide | 302.1183 | 1.5 | [M+Na]+ | 0.5 | 3.2 |

*Fold change: Fold change is calculated as the ratio of AIN-76A+DSS versus AIN-76A and BRB+DSS versus AIN-76A+DSS.


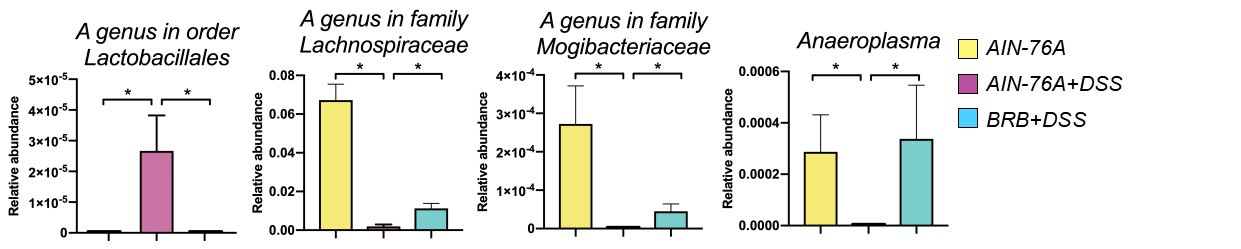


**Fig. S2.** Relative abundances of gut bacterial genera that were restored by BRB dietary intervention, including a genus in order Lactobacillales, a genus in family Lachnospiraceae, a genus in family Mogibacteriaceae, and *Anaeroplasma*. (AIN-76A, n=10; AIN-76A+DSS, n=9; BRB+DSS, n=10; *p<0.05).


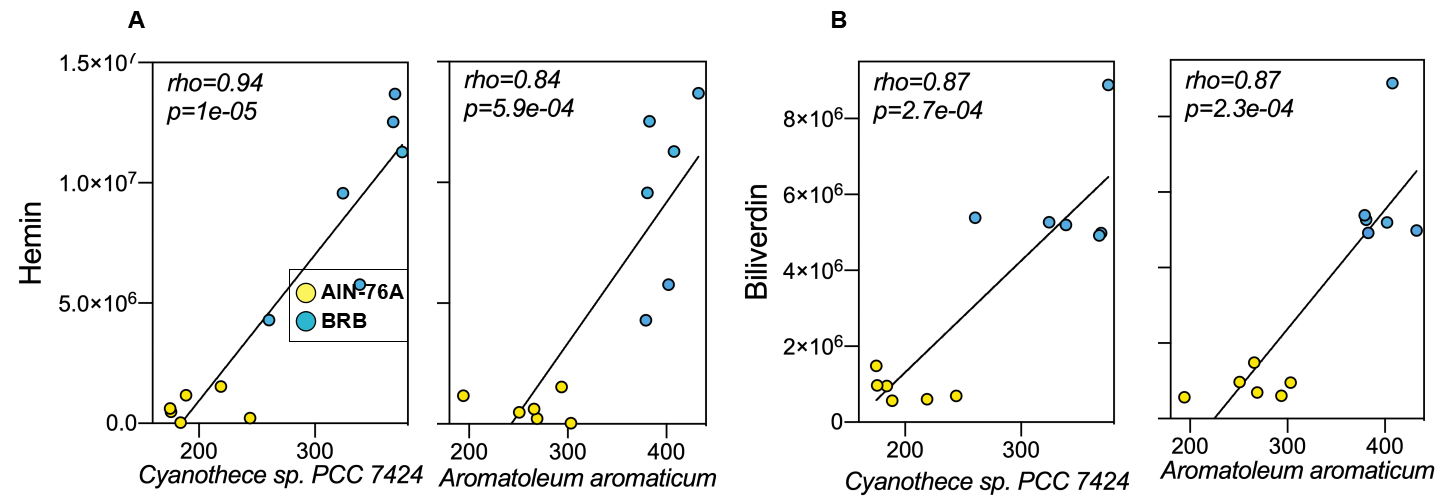


**Fig. S3.** Two bacterial species (**A**. *Cyanothece sp. PCC 7424*; **B.** *Aromatoleum aromaticum*) are significantly correlated with levels of both hemin and biliverdin (n=6; rho>0.8; p<0.001).

Table S2. Primers for quantatative RT-PCR.

| Gene | Forward primer | Reverse primer | Reference |
| --- | --- | --- | --- |
| TNF-α | CCCTCACACTCAGATCATCTTCT | GCTACGACGTGGGCTACAG | Alkhouri et al., 2010 |
| IL-6 | TAGTCCTTCCTACCCCAATTTCC | TTGGTCCTTAGCCACTCCTTC | Alkhouri et al., 2010 |
| IL-1β | GCAACTGTTCCTGAACTCAACT | ATCTTTTGGGGTCCGTCAACT | Alkhouri et al., 2010 |
| β-actin | CGTGCGTGACATCAAAGAGAA | TGGATGCCACAGGATTCCAT | Song et al., 2015; |

**References**

Alkhouri, N., Gornicka, A., Berk, M. P., Thapaliya, S., Dixon, L. J., Kashyap, S., . . . Feldstein, A. E. (2010). Adipocyte apoptosis, a link between obesity, insulin resistance, and hepatic steatosis. J Biol Chem, 285(5), 3428-3438. doi:10.1074/jbc.M109.074252

Song, J. M., Qian, X., Molla, K., Teferi, F., Upadhyaya, P., G, O. S., . . . Kassie, F. (2015). Combinations of indole-3-carbinol and silibinin suppress inflammation-driven mouse lung tumorigenesis by modulating critical cell cycle regulators. Carcinogenesis, 36(6), 666-675. doi:10.1093/carcin/bgv054
